# A Comparison of Corn (*Zea mays* L.) Residue and Its Biochar on Soil C and Plant Growth

**DOI:** 10.1371/journal.pone.0121006

**Published:** 2015-04-02

**Authors:** Francisco J. Calderón, Joseph Benjamin, Merle F. Vigil

**Affiliations:** USDA-ARS, 40335 Co Rd GG., Central Great Plains Research Station, Akron, Colorado, United States of America; Old Dominion Univ., UNITED STATES

## Abstract

In order to properly determine the value of charring crop residues, the C use efficiency and effects on crop performance of biochar needs to be compared to the un-charred crop residues. In this study we compared the addition of corn stalks to soil, with equivalent additions of charred (300 °C and 500 °C) corn residues. Two experiments were conducted: a long term laboratory mineralization, and a growth chamber trial with proso millet plants. In the laboratory, we measured soil mineral N dynamics, C use efficiency, and soil organic matter (SOM) chemical changes via infrared spectroscopy. The 300 °C biochar decreased plant biomass relative to a nothing added control. The 500°C biochar had little to no effect on plant biomass. With incubation we measured lower soil NO_3_ content in the corn stalk treatment than in the biochar-amended soils, suggesting that the millet growth reduction in the stalk treatment was mainly driven by N limitation, whereas other factors contributed to the biomass yield reductions in the biochar treatments. Corn stalks had a C sequestration use efficiency of up to 0.26, but charring enhanced C sequestration to values that ranged from 0.64 to 1.0. Infrared spectroscopy of the soils as they mineralized showed that absorbance at 3400, 2925-2850, 1737 cm^-1^, and 1656 cm^-1^ decreased during the incubation and can be regarded as labile SOM, corn residue, or biochar bands. Absorbances near 1600, 1500-1420, and 1345 cm^-1^ represented the more refractory SOM moieties. Our results show that adding crop residue biochar to soil is a sound C sequestration technology compared to letting the crop residues decompose in the field. This is because the resistance to decomposition of the chars after soil amendment offsets any C losses during charring of the crop residues.

## Introduction

Biochar is a heterogeneous material generated by the heating of biomass under limiting oxygen. Biochar comes in many forms, depending on the pyrolysis conditions and the type of feedstock [1,2]. Increases in pyrolysis temperatures result in progressively carbon-rich and recalcitrant organic materials [2]. Chars generated at low temperature tend to be higher in volatile compounds, while highly carbonized chars have lower CEC and tend to be more stable. In general, acid functional groups decrease and ash content increases with charring temperature.

Biochar could be particularly useful for increasing soil C beyond what conservation agricultural practices can achieve [3]. This is because biochar decomposes very slowly relative to soil organic matter (SOM) and plant residues [4]. Biochar, however, can have a priming effect on SOM, and can also result in temporary N immobilization, so the C sequestration value of each biochar needs to be evaluated and weighed against these possible drawbacks.

Diffuse reflectance IR spectroscopy (DRIFTS), also known as FT-IR, is now used to characterize and quantify biochar and soil pyrogenic C [1,5]. DRIFTS spectra contain qualitative information of important functional groups related to aromatics, phenolics, polysaccharides, and aliphatics among others [6]. Early in the charring process, the feedstock loses proportionately more O than C, and aliphatic functional groups are lost or converted to aromatics [2]. Bands for aliphatic C-H, C-O, and O-H, decrease in absorbance, while aromatic bands become more prominent in charred biomass [7]. Because of this, biochar signals can be detected in soils, with lower temperature biochars accentuating soil carbonyl and aliphatic bands, while higher temperature chars exhibit more aromatic absorbance [8]. However the DRIFTS spectra of highly carbonized chars do not have large mid infrared peaks due to the low dipole moment of the more prevalent functional groups.

Biochar can affect crop performance positively or negatively depending primarily on the feedstock type, but also the application rate, and pyrolysis conditions [9]. Biochar can be a particularly good amendment for tropical soils [10], because it can raise the pH of highly weathered soils and ameliorate Ca, Mg, and K deficiencies [11]. Biochar can enhance plant growth and N fertilizer use efficiency by remedying physical soil issues like those of hard setting alfisols [12], and improve the water-holding capacity of coarse soils [13]. The benefits of biochar due to base content, water holding capacity, and physical limitations may be less applicable to the mollisols of the high plains in the US wheat belt, given that these soils are calcareous, rich in K and Ca, and have a naturally high water holding capacity. Because of this, the effects of biochar amendment on crop performance in high plains soils needs to be quantified.

Charring removes both C and N from the feedstocks as the material is pyrolized, resulting in altered C to N ratios. Higher C to N ratios are thought to be associated with increased N demand upon decomposition. Biochar can limit crop growth on the short term due to N immobilization, and can reduce N_2_O fluxes by limiting N mineralization or by immobilizing N through adsorption [14]. N immobilization can be useful because it can reduce N leaching, and enhance N retention in agricultural soils. However, N immobilization can result in N deficiency in the crops immediately following application, unless the N fertilizer rates are adjusted to counteract the immobilization [15].

Previous studies have shown that charred bamboo leaf litter results in reduced CO_2_ emissions upon addition to soil relative to the un-charred leaf litter [16,17]. Because of the more recalcitrant nature of the bamboo biochar compared to the feedstock, the deployment of biochar is more beneficial than the fresh litter in a C sequestration context.In order to determine the effectiveness of biochar on C resistance to decomposition and N cycling dynamics, it is important to compare soil additions of biochars generated at different temperatures, as well as their feedstock. This way it can be determined if it is beneficial to char crop residues before incorporating them into the field, as opposed to simply incorporating the residue and let it decompose naturally. In this study we carried out a laboratory incubation of two temperature biochars and their corn stalk feedstock mixed with two different soils. We measured mineral N dynamics, total C and N, and DRIFTS on the soil-char mixes as they mineralized. DRIFTS was used to study the biochar and SOM chemical attributes that are related to net N mineralization and C loss, and to measure qualitative changes in SOM during mineralization with biochars, feedstock, and soil alone. We also carried out a growth chamber experiment in which we grew plants in soils amended with the chars and feedstock, in order to determine their effect on plant growth and N content.

We tested the hypotheses that 1) charred material will have higher C sequestration efficiency than the feedstock, and 2) that adding char to soil will cause less plant N deficiency relative to the corn stalk feedstock. We will try to answer the questions: 1) Does charring temperature affect corn stalk C’s resistance to decomposition? 2) What will result in more C sequestration, biochar or an equivalent amount of uncharred corn feedstock? 3) Will adding char affect N availability to the growing crop?

## Materials and Methods

### Soils used in the study

The soils (0–20 cm) were obtained at the Central Great Plains Research Station in Akron, Colorado, U.S.A. The location of the study (40.15 deg. N latitude and 103.15 deg. W longitude) is a field research station where the three authors of the study are based. Beacuse of this, specific permissions were not required for this location or activities. The field studies did not involve endangered or protected species. Both soils are silt loams in the Weld series (Aridic Paleustolls), obtained from adjacent fields. The non-eroded soil was obtained from a field under grass, and the eroded soil was obtained from an adjacent site that had been mechanically eroded down to the B horizon. The soils varied in their pH and Ca content due to the presence of a calcium carbonate hardpan closer to the surface of the eroded soils (Table 1). The soils were air dried and sieved free of pebbles and plant residue using a 2 mm sieve.

**Table 1:** Texture, pH and base content of the soils used in the study.

		High-erosion soil	Low-erosion soil
Sol. Salts	mmhos/cm	0.74	0.79
Bicarbonate. P	mg kg^-1^	20	32
K*	mg kg^-1^	819	820
Ca*	mg kg^-1^	2685	1795
Mg*	mg kg^-1^	375	393
Na*	mg kg^-1^	24.5	27.5
CEC	me/100g	18.8	17.5
sand	%	31.0	29.0
silt	%	62.0	55.5
clay	%	7.0	15.5

*- NH_4_OAc (Exchangeable).

### Production of the biochars

Corn stalks were obtained from the field after corn senescence and harvest. The corn stalk material was dried (60 °C), and shredded before the charring. The biochars were produced using a method detailed in [18]. The shredded stalks were packed in 125 mL Erlenmeyer flasks, and the head space at the top was packed with glass wool. The Erlenmeyers were then capped loosely with a 50 mL beaker. The stalks were then subjected to the 300 or 500 °C temperatures for 45 min in a muffle furnace, then brought to room temperature. The stalks lost 46.3 percent of their mass at 300 °C, and 69.8 percent at 500 °C. The uncharred stalks, 300 °C biochar, and 500 °C biochar were 43.3, 57.9 and 69.8 percent C respectively. The biochar C content falls within the range reported by others for biochars made from corn stover feedstock and similar pyrolysis temperatures [9]. The 300 °C and 500 °C biochars had a pH of 6.3 and 9.6 respectively (Table 2).

**Table 2:** pH of the soils and soil-biochar mixtures.

Treatment	pH
300° C biochar alone	6.3
500° C biochar alone	9.6
	High-erosion soil and mixtures
Control (soil alone)	7.5
Soil plus corn stalks	6.5
Soil plus 300° C biochar	7.5
Soil plus 500° C biochar	7.6
	Low-erosion soil and mixtures
Control (soil alone)	5.2
Soil plus corn stalks	5.5
Soil plus 300° C biochar	5.5
Soil plus 500° C biochar	5.8

### Incubation conditions

Before the incubation, the biochar plus soil mixtures were placed in tubes then packed lightly. Screw-capped 50 mL test tubes containing 25 g of sieved dry soil (60 °C) were used for the incubations. We incubated the following four types of mixtures, which constitute the biochar treatments: 1) The two different temperature biochars (ground with a mortar and pestle before the mixing). 2) Corn stalk residue (ground with a retsch grinder). 3) A nothing-added control treatment. We added the biochar or stalks to each tube at a rate of 12.5 g kg^−1^ soil equivalent to a field incorporation of 20 t stalks ha^−1^ into the top 12.7 cm of soil. We added the stalk or biochar on a per un-charred stalk weight basis, so the tubes receiving char received less than 12.5 g kg^−1^, depending on the percent weight loss upon charring. This was 6.7 g kg^−1^ of 300 °C biochar, and 3.8 g kg^−1^ of 500 °C biochar. At time zero the soils were brought to 10 percent gravimetric water content with distilled water. We added one drop from a 2:1 water:soil filtrate from the 0–5 cm depth of a fallow organic wheat site in order to inoculate the tubes. Destructive samplings were carried out at 0, 2, 6, 10, 14, 20, and 48 weeks. At each time we sampled 10 tubes per soil/biochar treatment combination. Of those ten replicates, all were extracted for mineral N and scanned for DRIFTS, but only four replicates were analyzed for total C and N. The total C and N was analyzed with at LECO Truspec analyzer (LECO Corporation, St. Joseph, Michigan). C sequestration efficiency during the incubation was calculated as: (increased soil C relative to the control at the end of the incubation)/(added C at time zero). The mineral N in the soils was analyzed on extracts of 10 g of soil in 25 ml 2M KCl using a LACHAT Quickchem flow injection analyzer (Lachat Instruments, Loveland, Colorado), and the data was calculated as oven dry basis.

The pH of the soil-biochar mixtures was measured by mixing 20 g of sample with 20 mL 0.01M CaCl_2_. The biochars alone were mixed at a ratio of 1g biochar to 20 mL solution. The samples were then stirred for ½ hour, then let sit sit for another ½ hour undisturbed. At this point the pH was determined with a Horiba d-24 ph/orp-conductivity meter (Horiba, Kyoto, Japan).

The soils were dried at 60 °C and ground using a mortar and pestle before DRIFTS analysis. Scans were done in diffuse reflectance from 4000 to 400 cm^−1^ on neat (undiluted) samples using a Digilab FTS 7000 (Varian Inc., now Agilent Technologies) with a DTGS detector and a KBr beam splitter. KBr was used as background, which was subtracted from each recorded spectrum. Sixty four scans were co-added at 4 cm^−1^ resolution for each sample.

### Growth chamber experiment

Five kg of dried and sieved high-erosion soil was used per pot. We used the high-erosion soil in order to imitate char addition to an eroded low productivity soil, which is one of the likely scenarios for biochar deployment in the field. The biochar treatments consisted of corn stalk, 300^o^ char, 500^o^ char, and nothing added (control), with 5 replicates per treatment. The added amendments were mixed with the top 15.2 cm of soil to simulate incorporation by field tillage. The soil in the pots received the equivalent of 12.6g Kg^-1^ of corn stalks, and the amount of biochar was adjusted according to the weight loss during charring as explained for the incubation experiment. Four proso millet (*Panicum miliaceum*) seeds were planted per pot. All plants received 13 mg per plant of N as KNO_3_ the second week of growth, comparable to a 67 Kg ha^-1^ application. The plants were grown for 6 weeks in a growth chamber with a 16h photoperiod, at 60 percent relative humidity, and constant 22 °C temperature. The harvest was done before the plants became pot bound, previous to the reproductive stage 68 d after planting. At harvest, the plant biomass per pot was recorded, and the plant tissues were dried and ground before total C and N analysis on a LECO Truspec (Leco, St. Joseph, Michigan).

### Statistical Analyses

The ANOVA for the incubation experiment were carried out using the PROC MIXED analysis of SAS Release 8.02 (SAS Institute, Cary, NC). Mean separations were done with Duncan’s Multiple Range tests. The Shapiro-Wilk statistic was used to test the normality of the data.

Spectral subtractions and spectral smoothing were done with GRAMS AI version 9.1 (Thermo Fisher Scientific Inc.). We used the default subtraction settings which include the factor, increment, tolerance and autoscaling parameters. The smoothing was done with the Savitzky Golay 2^nd^ polynomial and 20 point convolution function.

## Results

### Charring effect on corn stalk chemistry

**Fig 1:**
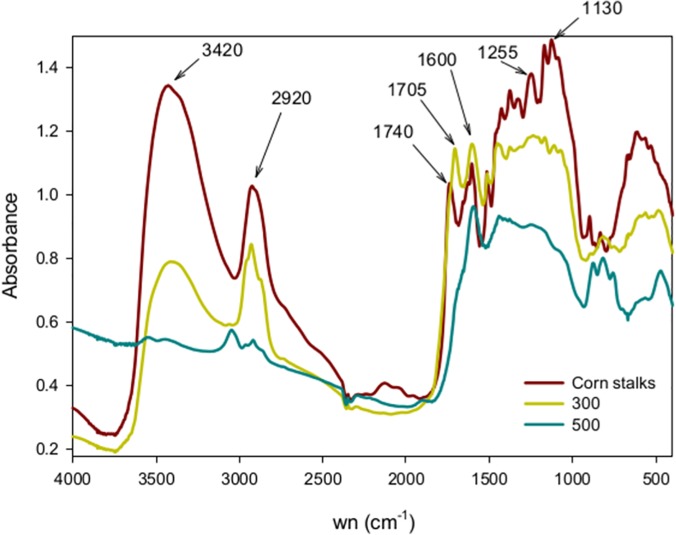
Average spectra of the corn stalks, 300 degree char, and 500 degree char. wn = wavenumbers in cm^-1^.

Both the percent C and percent N content of the corn feedstock increased as it was charred, but proportionately more C than N was lost, resulting in biochars with lower C to N ratio relative to the feedstock. The corn stalks had a percent C, percent N, and C to N ratio of 43.3%, 0.91%, and 47.5 respectively. The 300 °C biochar had values of 57.9%, 1.5%, and 39.7. The 500 °C biochar had values of 69.8%, 1.6% and 43.9 respectively. Fig 1 shows the DRIFTS spectra of the stalk feedstock and the biochars produced from it. All biochar functional group assignments are as in Parikh et al. [6]. The charring at 300 °C resulted in the loss of oxygen containing functional groups like OH/NH (3420 cm^-1^) and C = O (1740 cm^-1^). The 300 °C biochar has a aromatic carbonyl/carboxyl C = O peak at 1705 cm^-1^ that was absent in the corn feedstock. The 500 °C charring further removed aliphatic CH absorbance (2920 cm^-1^), phenolic C-O (1255 cm^-1^) and polysaccharide C-O (1130 cm^-1^). The bands at 3050 cm^-1^ and 1600 cm^-1^ mark the presence of aromatic CH and OH created between 300 and 500 °C.

### Effects of biochar and feedstock on plant performance

Addition of the 300 °C char and the uncharrred feedstock reduced proso millet biomass relative to the controls (Table 3). The lowest biomass values were recorded on the plants receiving corn stalk feedstocks. The 500 °C char was marginally lower than the control (p = 0.07). In contrast, tissue N was highest in the plants that received the corn stalks and the chars relative to the controls (Table 3). Total plant N uptake (not shown) was significantly higher in the controls than the stalks and chars due to the markedly higher plant biomass, even with the lower tissue N concentration in the controls. Plant tissue C to N ratios were also highest in the controls relative to the biochar and feedstock amendments.

**Table 3:** Mean proso millet biomass, tissue C, tissue N, and C to N ratio at harvest.

Treatment	Dry biomass (g)	N (g kg^-1^)	C (g kg^-1^)	C to N ratio
Control	4.55(1.93)^a^	20.0(5.2)^c^	406.8(7.0)^a^	20.4(6.0)^a^
Corn stalks	1.91(1.39)^b^	27.1(5.1)^ab^	396.3(11.0)^bc^	14.6(3.34)^cd^
300° C	2.16(1.30)^b^	27.6(3.1)^a^	391.6(6.5)^c^	14.2(2.13)^d^
500° C	2.91(1.19)^b^	24.4(2.0)^bc^	398.8(4.1)^b^	16.3(1.58)^bc^

Numbers not sharing a letter suffix within a column are different according to a T test (p<0.05). n = 5. The standard deviations are in parenthesis.

### Laboratory incubation mineral N dynamics

**Fig 2:**
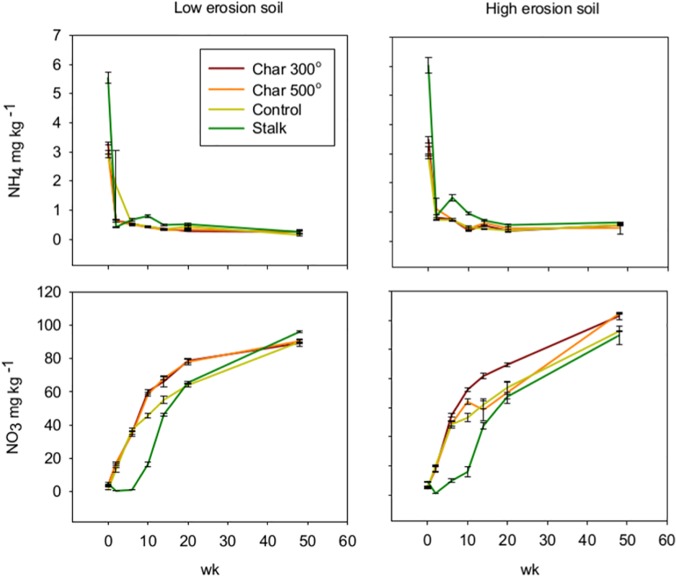
Mineral N dynamics during the incubation of the low-erosion (right graphs) and high-erosion soils (left graphs). Ammonium data is in the top graphs, and nitrate data is in the bottom graphs. Error bars are the SEM.

The ANOVA results indicate that there were significant (p< 0.001) incubation time and biochar treatment effects on NH_4_ and NO_3_ for both high-erosion and low-erosion soils (Table 4). The mineral N dynamics were very similar for the high and low-erosion soils, indicating that soil NH_4_ and NO_3_ were mainly determined by biochar treatment and secondarily by soil type (Fig 2). In both soils, Duncan’s Multiple Range test mean separations (p< 0.05) indicate that NH_4_ was significantly higher in week zero than the rest of the incubation times for all biochar treatments, and significantly higher in the Stalk treatment. Nitrification activity was strong in both soils given the steep drop in NH_4_ after week 0 in all treatments, although there was a small rise in the stalk treatment between weeks 6 and 10 (Fig 2). The NO_3_ data shows a time effect due to the buildup of NO_3_ during the incubation. The Duncan’s Multiple Range test shows that in the low-erosion soil, the NO_3_ contents of the 300 °C and 500 °C chars were higher than the Stalk, which in turn was higher than the Control. Fig 2 shows that the biochar amended soils had higher NO_3_ between approximately 10–30 weeks of incubation time. In the high-erosion soils, the NO_3_ was 300 °C > (500 °C, Control) > Stalk.

**Table 4:** Analysis of Variance results of the generalized linear mixed models for the incubated high-erosion and low-erosion soils.

Low-erosion				
Effect	NO_3_		NH_4_	
	F-value	p-value	F-value	p-value
Incubation time	1701.58	<.0001	89.16	<.0001
Biochar treatment	174.51	<.0001	5.47	0.001
Incubation x biochar	39.25	<.0001	4.58	<.0001
**High-erosion**				
Effect	NO_3_		NH_4_	
	F-value	p-value	F-value	p-value
Incubation time	389.23	<.0001	463.36	<.0001
Biochar treatment	66.07	<.0001	38.88	<.0001
Incubation x biochar	12.07	<.0001	20.34	<.0001

Effects of biochar type, and incubation time are shown.

### Laboratory incubation soil total C and total N dynamics

**Fig 3:**
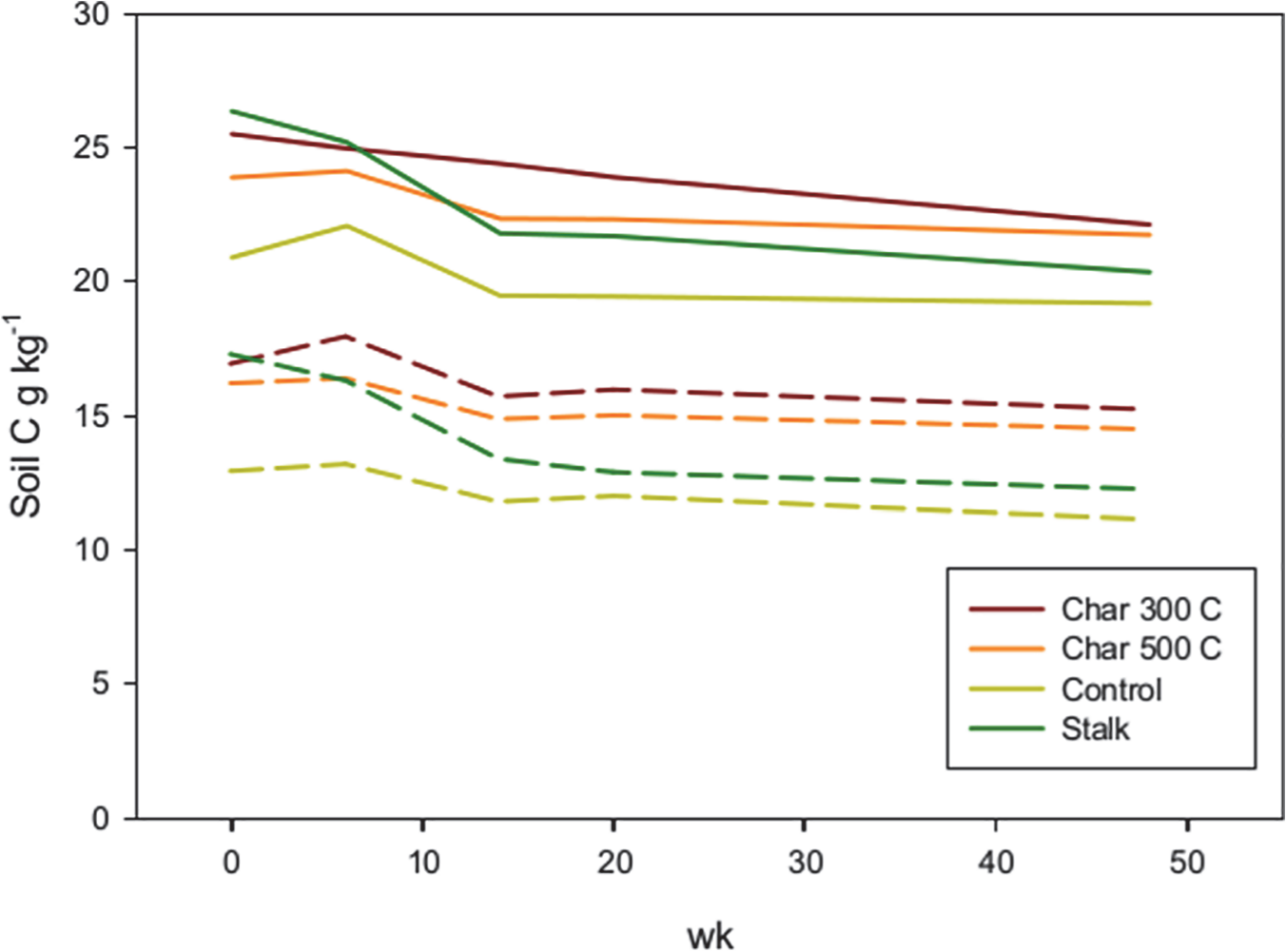
Soil C dynamics during the incubation of the low-erosion (dashed lines) and high-erosion soils (solid lines).

The soil C had significant incubation time and biochar amendment main effects in both soils according to ANOVA (p> 0.001). Over the 48 week incubation the soil C declined by 13.7% in the high-erosion soils and by 16.2% in the low-erosion soils (Table 5). In both low and high-erosion soils, mean separations shows that total C in weeks 0 and 6 are significantly higher than weeks 14 and 20, which in turn are significantly higher than week 48 (p< 0.05) (Fig 3). Duncan’s Multiple Range tests shows that total soil C in the biochar treatments are significantly different from each other in both soils: 300 °C > (500 °C, Stalk) > Control.

**Table 5:** Total soil C, N, C/N and C sequestration efficiency of the control soils and soil amended with corn stalks, 300 °C char, and 500 °C char before the incubation and at the end of the incubation.

			High-erosion Soil				
	Nitrogen		Carbon		C/N		C sequestration
	0 weeks	48 weeks	0 weeks	48 weeks	0 weeks	48 weeks	efficiency
Biochar 300 °C	1.44(0.06)	1.42(0.07)	25.5(2.01)	22.1(0.70)	17.69(0.99)	15.63(0.28)	0.64
Biochar 500 °C	1.39(0.04)	1.39(0.09)	23.9(0.73)	21.7(1.22)	17.19(0.32)	15.67(0.19)	0.85
Control	1.21(0.08)	1.31(0.01)	20.9(0.44)	19.2(0.31)	17.28(1.32)	14.63(0.33)	NA
Stalk	1.48(0.09)	1.44(0.06)	26.3(1.40)	20.3(0.14)	17.85(0.68)	14.17(0.47)	0.21
			Low-erosion Soil				
	Nitrogen		Carbon		C/N		
	0 weeks	48 weeks	0 weeks	48 weeks	0 weeks	48 weeks	
Biochar 300 °C	1.70(0.03)	1.73(0.10)	16.9(0.09)	15.2(1.77)	9.96(0.36)	8.80(0.52)	1.02
Biochar 500 °C	1.70(0.06)	1.76(0.08)	16.2(0.74)	14.5(0.83)	9.52(0.12)	8.24(0.10)	1.02
Control	1.52(0.07)	1.53(0.11)	12.9(0.36)	11.1(0.37)	8.51(0.33)	7.29(0.28)	NA
Stalk	1.66(0.03)	1.68(0.00)	17.3(0.50)	12.3(0.15)	10.43(0.33)	7.28(0.11)	0.26

C and N data is in g kg^-1^. NA = not applicable.

Different letter suffixes within a column are significantly different (p< 0.05) according to a T test. Standard deviations are in parenthesis.

Total soil N was statistically indistinguishable between week 0 and week 48 in both the high-erosion and low-erosion soils (Table 5). There was a biochar treatment main effect with the 300 °C > (500 °C, Stalk) > Control in the low-erosion soil, and the 300 °C > 500 °C > Stalk > Control in the high-erosion treatment (p< 0.001).

Despite the lack of a net change in total soil N during the incubation, there was an average reduction in C to N ratio of 14.2% and 17.7% in the high-erosion soil and low-erosion soil respectively (Table 5). This is explained by the decline in total C during the incubation (Fig 3).

The C sequestration efficiency of the biochars was significantly higher than the corn stalk feedstock in both soils according to a student’s T-test (*p*< 0.01) (Table 5). The C sequestration efficiency of the 500 °C and 300 °C biochars was statistically indistinguishable. The soil type affected the C sequestration efficiency of the chars, with the High-erosion soil having values of 0.64–0.85, and the Low-erosion soil having values 1.0 for both chars (Table 5).

### Laboratory incubation SOM chemistry and DRIFTS analysis

**Fig 4:**
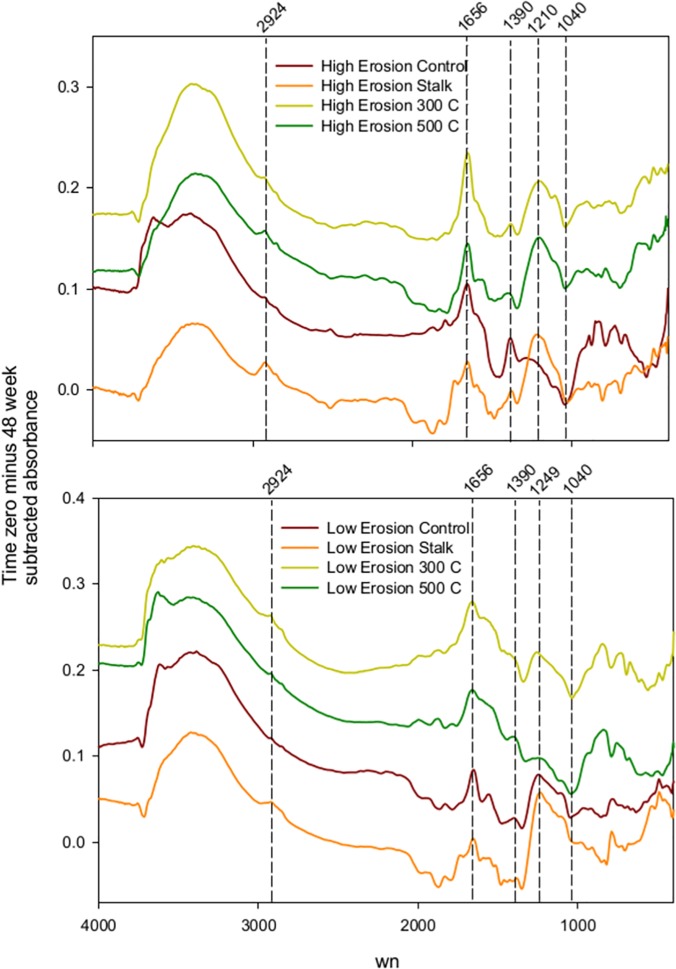
Spectral subtraction of the mid infrared spectra from the unamended soil controls, soil plus stalk feedstock, soil plus 300 degree biochar, and soil plus 500 degree biochar treatments. Subtractions are time zero spectra minus 48 wk incubation soil spectra. Top graph is the High-erosion Soil, and bottom graph is the Low-erosion Soil. wn = wavenumbers in cm^-1^.

We carried out spectral subtractions of the time zero minus 40 week incubated soils to determine how SOM chemistry changes as it mineralizes in the presence or absence of biochar or corn feedstock (Fig 4). Both high-erosion and low-erosion soils lost absorbance at the broad band around 3400 cm^-1^ for OH/NH stretching and the aliphatic CH bands between 2925–2850 cm^-1^ [6]. The absorbance loss at 2925–2850 cm^-1^ was more marked with the stalk treatment. The subtracted spectrum for the stalks forms a shoulder at 1737 cm^-1^ for carbonyl C = O (Fig 4). All soils had a marked reduction in absorbance at 1656 cm^-1^, a band attributed to amide C = O stretch, but also close to the aromatic C = C band between 1650–1600 [6]. The decreased absorbance at 1390 cm^-1^ is due to loss of carboxylate or phenolic C-O functional groups. Reductions in the 1249 cm^-1^ band, which are marked in the Stalk treatment, correspond to a combination of carboxylic acid C-O stretch, OH deformation, and ester, or phenol C-O stretch. Absorbance at 1040 cm^-1^ increased during the incubation in several treatments. This band is within the polysaccharide C-O stretch region, but could also be due to Si-O stretch (Fig 4).

**Fig 5:**
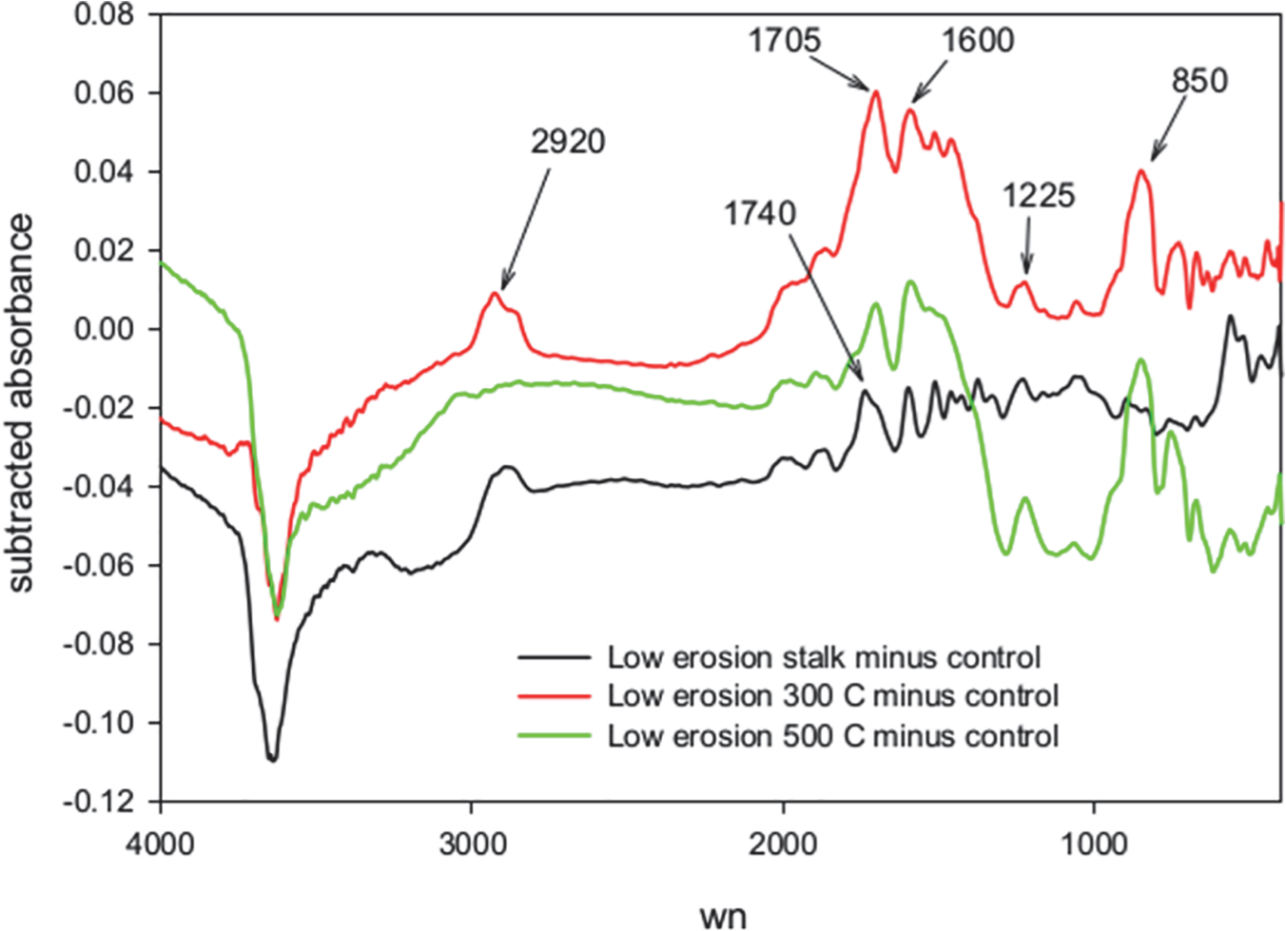
Spectral subtraction of the time zero and 48 week incubated soil-char mixes for the eroded and non-eroded soils. Soil amendments were corn stalks, 300 °C char and 500 °C char. wn = wavenumbers in cm^-1^.

Spectral subtractions of the biochar-amended soils and the controls at time zero illustrate the spectral differences caused by the char additions (Fig 5). Overall, the amendments caused relatively small but predictable changes in the soil spectra. Adding the stalk feedstock to the low-erosion soil resulted in increased absorbance at several bands present in the stalks alone: 3300, 2960–2830 cm^-1^, 1740, and 1600 cm^-1^. The 300 °C biochar addition increased absorbance at 2960–2830 cm^-1^, 1705, 1600, 1510, 1460, 1225, and 850 cm^-1^.The most prominent subtractive peaks occur at 2920, 1705, and 1600 cm^-1^, which are among the most marked features of the 300 °C biochar spectum (Fig 1). In contrast with the 300 °C biochar, the 500 °C biochar subtractive spectrum does not have the aliphatic CH band at 2960–2830 cm^-1^, consistent with the 500 °C pure biochar spectrum. All subtractive spectra had a negative peak 3645 cm^-1^ where lattice clays absorb.

## Discussion

### Charring effect on corn stalk chemistry

The 300 °C and 500°C conditions produced biochars with markedly different chemical makeup. The 300 °C biochar spectra are consistent with a material that has been torrefied but still contains many of the spectral features of the stalk feedstock. DRIFTS has been used to illustrate how charring results in higher aromatic absorbance, and eventually the loss of spectral features due to the formation of a graphite-like substance [18]. The changes in the 1705–1740 cm^-1^ region suggest the formation of carbonyls from cellulose dehydration [1]. The loss of absorbance at 3400 and 2920 cm^-1^ are the result of the removal of thermolabile OH/NH and aliphatic CH functional groups. The 500 °C biochar in turn has lost many of the bands in the original feedstock and shows signs of higher aromaticity, but is not yet highly carbonized. Reeves et al. [1] observed that spectral bands in the 1500–1000 cm^-1^ fingerprint region are lost in high temperature chars, but in this study we still observe aromatic bands in the 500 °C biochar. The peak at 1600 cm^-1^ in the 500 °C biochar can be due to quinones formed during the pyrolysis of cellulose [1].

### Plant Biomass at harvest

Biochar amendments are not always associated with crop yield increases upon field application [9,11,19–21]. Plant biomass responses to biochar addition have shown great variability due to the many combinations of feedstock quality, pyrolysis conditions, biochar application rates, crop demands, and soil types [9,11]. Nevertheless, corn stalk biochars from a range of temperatures were shown by [9] to enhance corn yields.

In this study, the reduced plant biomass on the un-charred Stalks treatment can be explained by microbial N immobilization, which can be exacerbated by amendments with a high C to N ratio [22,23]. The N immobilization by the stalks is supported by lower soil NO_3_ concentrations during the incubation experiment. The 300 °C biochar also caused reduced millet biomass relative to the control, but the incubation mineral N data, as well as the high tissue N of the plants grown in the 300 °C char indicate that other factors besides N immobilization may be at play.

The reduced growth of the millet in the 300 °C char suggests a non-nutritional issue such as char toxicity, Na issues, or pH. Biochar sodium can reduce plant growth [9]. However, corn stover tends to produce chars of relatively low Na content relative to other feedstocks, and the soils used in this study were moderately low in Na (< 27.5 mg kg^-1^), suggesting that Na is not likely the problem in this case. Furthermore, Na would have been added in the same amounts with the feedstock, as with the chars. Biochars could also reduce crop growth due to increased pH [19]. Both biochars had a small liming effect on the high-erosion soil (Table 2), but the resulting 7.5–7.6 pH of the soil-biochar mixtures was not likely to have a marked detrimental effect on the millet biomass.

Biochar rates higher than 20 t ha^-1^can result in decreased crop growth [9,19], but the rates used in this experiment were comparable to field applications of 10.6 and 6.0 t ha^−1^ for the 300 and 500 °C biochars respectively. The 500 °C biochar treatment produced higher millet biomass than the 300 °C. Rajkovich et al. [9] observed that increasing the pyrolysis temperature of certain types of feedstocks reduced the negative effects of the chars on crop growth, but this was not the case with all biochars. Higher temperature chars tend to have less volatile matter, and form aromatic and heterocyclic structures which resist chemical attack by microbes [24], consistent with the DRIFTS and C sequestration efficiencies observed in this study. It has been hypothesized that labile C in chars decreases with charring temperature. The more stable high temperature chars will cause lower reductions in plant growth due to reduced competition for N with soil microbes [9].

The addition of 300 °C biochar resulted in high tissue N in the millet, but less so in the 500 °C biochar. The lower tissue C to N in the proso millet amended with the corn stalk and chars has implications for forage quality and millet residue decomposability.

### Incubation mineral N dynamics

Biochars can have large differences in C to N ratios, and values ranging from 7–400 have been reported [25]. A C to N ratio of of approximately 20 is thought to be a threshold for N immobilization in organic materials, but little is known how this relates to biochars. The values of our feedstock and two biochars ranged from 47.5 to 39.7, suggesting that these materials should all cause net N immobilization upon addition to soil. Biochar ammendment has been shown to cause soil N immobilization and decrease foliar tissue N in crops [9]. In this study, however, we found that the 300 °C biochar resulted in higher shoot tissue N than the control. High tissue N concomitant with low biomass tends to be a symptom of drought, but in this study all biochar and control treatments were watered equally, suggesting that other sources of stress might have been responsible.

Results on the effect of biochars on mineral N have been varied. Biochars can reduce the soil N available to plants both by microbial immobilization and by adsorption of soil mineral N [15,26]. However, certain biochars can have a positive effect on soil mineral N. For example, Prendergast-Miller et al. [27] found that hardwood biochar can produce elevated rhizosphere NO_3_. Corn residue has been known to cause ephemeral net N immobilization in soils [22]. In our incubation experiment, corn stalks caused a decline in soil nitrate levels that lasted between 10 and 20 weeks. This is an important time frame given that many summer crops have their highest N demands during the vegetative growth stage when biomass growth rates are the fastest. While both biochars and the corn Stalk amendments had C to N ratios that were expected to produce N immobilization, only the Stalk treatment showed a period of nitrate decline relative to the control soil. The biochars had a positive effect on soil nitrate in both soils, suggesting that they contained mineralizable N, which can add to the plant available N pool. Mineralizable N tends to be higher in lower temperature chars because the presence of amino acids and other labile N compounds [28]. Others have found that low temperature chars may contain enough labile C to promote microbial growth and N immobilization [29]. In this study, however, we show that the 300 °C char, which showed a measureable amount of C mineralization during the incubation, was not associated with decreased soil NO_3_.

### Incubation soil C and N dynamics

The stalk feedstock, with a C sequestration use efficiency of 0.21–0.26 shows that the majority of the C contained in corn residue is prone to mineralization and loss from the soil. Charring considerably improved C sequestration to values that ranged from 0.64 to 1.0. Soil type had an effect on the biochar C sequestration efficiency, with the low-erosion soils having values of 1.0 for both biochars. Our study based the C sequestration efficiency on a C mass balance relative to the controls, rather than isotope tracer work. Assuming that some of the C mineralized in the low-erosion soil was from the biochar, results suggests that the addition of both biochars to the low-erosion soils was associated with decreased soil C mineralization, also referred to as negative priming. Our results contrast with previous work indicating that negative priming is more likely on long incubations with biochars made at high temperatures and from hardwood feedstocks [30].

Work by others has shown that char mineralization tends to decrease with increasing pyrolysis temperature [31], possibly because the formation of recalcitrant aromatic compounds [2]. Wang et al., [16] found that compared to the bamboo leaf feedstock, char addition to soil resulted in higher organic C storage after 12 months. They attribute the char’s effect to a reduced impact on microbial biomass, dissolved organic C, and CO_2_ fluxes relative to the feedstock. Wu et al. [17] Compared wheat straw feedstock with its biochar and found that comparatively up to 77% of the un-charred straw C was lost as CO_2_ upon incubation. The increased C loss from the straw was related to the higher dehydrogenase and β-glucosidase content of straw-ameded soils relative to biochar-amended soils.

Our results indicate that charring corn residue at 500 °C, then adding it back to soil results in a net sequestration of C. Although pyrolysis causes a mass loss of 70% of the feedstock C, the 500 °C char can reach nearly 4 times the C sequestration efficiency of the un-charred stalks.

### Incubation SOM dynamics and DRIFTS analysis

Previous work showed that addition of similar biochars to soil, in amounts enough to increase the soil total C by 50%, result in predictable changes in the soil DRIFTS spectra [8]. In this study, with increases in soil C ranging from 14–34%, spectral subtractions were able to resolve small differences between the time zero spectra belonging to the biochar and stalk soil mixtures and soil-alone controls. The subtractive spectra had bands associated with the added feedstocks or biochars. For example, the alipahtic CH band at 2960–2830 cm^-1^ was present in the 300 °C biochar and stalk subtraction, but not in the 500 °C subtraction. Increased absorbance at 1705 cm^-1^ in the biochar-amended soils corresponds to aromatic carbonyl or carboxyl C = O, while increases at 1600 cm^-1^ are consistent with skeletal C = C vibrations [6]. Absorbance at 850 (Aromatic C-H) and 1225 cm^-1^ (Carboxylic acid, ester,or phenol C-O, OH deformation) are not strong bands in the pure biochars, but give subtractive peaks. The inverted clay band in the subtractive spectra can be explained by the chars and feedstock amendments partially blocking the presentation of clays to the infrared detectors.

Subtractions of the spectral data before the incubation from after the incubation is meant to accentuate the spectral bands that are lost from soil as it decomposes. The control soils lost similar absorbance bands compared to the soils that received the biochars or feedstock. this suggests that the spectral changes observed during the incubation reflect largely the mineralization of SOM, and/or bands that are shared between the SOM and the biochar and feedstock amendments. Absorbance at 3400 and 2925–2850 cm^-1^, which are lost during the incubation, are associated with the soil light fraction C of low MRT [32], and also with changes in the protein and polysaccharide content of soil [8]. These bands may also have some contribution from the 300 °C biochar and feedstock. Absorbance at 3400 and 2925 cm^-1^ are lost from organic materials as they decompose in soils [33]. All this indicates that these bands represent the organics that are vulnerable to relatively rapid mineralization. The absorbance loss at 1737 cm^-1^ in the Stalk treatment may be indicating the decomposition of carbonyls in esters in the added corn material. Loss in absorbance at 1656 cm^-1^ is consistent with the mineralization of proteinaceous material in all soils, corroborated by the accumulation of mineral N during the incubation. Increased absorbance at 1040 cm^-1^ upon incubation could be explained by increased exposure and absorbance of silicates as organic matter decomposes in the soil. Absorbances near 1600, 1500–1420, and 1345 cm^-1^ were less affected by the mineralization, suggesting that they represent the refractory SOM moieties.

## Conclusions

Previous studies about biochar effects on crop growth have compared biochar-amended soils to nothing added controls. In this study, we compared the biochar to the feedstock, in order to test the benefits of charring residue and applying it back to the field.

Both the stalk feedstock and the low temperature char decreased proso millet biomass. This suggests that there is no penalty in terms of crop performance of charring crop residues relative to simply incorporating the same amount of residue. Charring crop residues does not increase N fertilizer requirements relative to the feedstock, but our results show that the 300 °C char did reduce plant biomass yield. There was a higher amount of net N mineralization in the biochar-amended soils compared to the stalks during the laboratory incubation, suggesting that the decrease in plant biomass is not entirely explained by N immobilization. Our results support the charring of crop residues as a sound C sequestration technology, given the large increases in C use efficiency of the charred material. We show that charring the corn stalks improves the C sequestration efficiency to the point that any C losses during the charring procedure are overcome.

The results of this study have to be viewed in light of the type of experiment that was undertaken. Any positive effects on plant growth that the biochars might have had by improving water use efficiency would have been negated by the watering regime used. In addition, any K, Ca, and Mg added with the chars would not have been of benefit to the millet plants given the calcareous nature of the high-erosion soil.
